# Distinct individual differences in default mode network connectivity relate to off-task thought and text memory during reading

**DOI:** 10.1038/s41598-019-52674-9

**Published:** 2019-11-07

**Authors:** Meichao Zhang, Nicola Savill, Daniel S. Margulies, Jonathan Smallwood, Elizabeth Jefferies

**Affiliations:** 1 Department of Psychology, University of York, Heslington, York YO10 5DD UK; 2 School of Psychological and Social Sciences, York St John University, York, YO31 7EX UK; 3 Frontlab, Centre National de la Recherche Scientifique (CNRS) UMR 7225, Institut du cerveau et de la moelle épinière (ICM), 75013 Paris, France

**Keywords:** Language, Reading

## Abstract

Often, as we read, we find ourselves thinking about something other than the text; this tendency to mind-wander is linked to poor comprehension and reduced subsequent memory for texts. Contemporary accounts argue that periods of off-task thought are related to the tendency for attention to be decoupled from external input. We used fMRI to understand the neural processes that underpin this phenomenon. First, we found that individuals with poorer text-based memory tend to show reduced recruitment of left middle temporal gyrus in response to orthographic input, within a region located at the intersection of default mode, dorsal attention and frontoparietal networks. Voxels within these networks were taken as seeds in a subsequent resting-state study. The default mode network region (i) had greater connectivity with medial prefrontal cortex, falling within the same network, for individuals with better text-based memory, and (ii) was more decoupled from medial visual regions in participants who mind-wandered more frequently. These findings suggest that stronger intrinsic connectivity within the default mode network is linked to better text processing, while reductions in default mode network coupling to the visual system may underpin individual variation in the tendency for our attention to become disengaged from what we are reading.

## Introduction

Despite the value of reading to our species, everyday experience indicates that even if we are reading for comprehension, our thoughts and feelings do not always remain tethered to the events that take place in the narrative. Indeed, multiple studies have demonstrated that off-task thinking while reading, often referred to as mind-wandering, is ubiquitous across cultures and has a measurable and reliable negative effect on reading comprehension (for examples, see^1–3^). Although the frequency and consequences of off-task thinking while reading are well-documented, we lack a clear understanding of the underlying neurocognitive architecture which gives rise to this experience.

Both off-task thought and reading are thought to depend on a complex component process architecture^4,5^. Reading involves the interaction of multiple cognitive processes^6–10^, including decoding and recognition of incoming perceptual information, semantic retrieval and discourse-level inference. The ventral visual stream, which supports increasingly abstract visual-to-semantic mappings within the temporal lobe, contributes to how we make sense of visual input^11–14^. Posterior temporal regions, such as the visual word form area, are implicated in orthographic processing^12,15^. More anterior regions of the temporal lobe exhibit a greater response to word meaning than to the visual properties of words^11,16,17^. Contemporary accounts of semantic cognition suggest that the most anterior aspects of the ventral anterior temporal lobes (ATL) represent heteromodal concepts that are formed from the convergence of multiple meaningful inputs across modalities^5,18,19^. Reading for comprehension also requires sustained attention to focus on orthographic information as the text unfolds word by word^20–25^, with the disruption of attentional mechanisms playing a causal role in the failure of comprehension^22^. Executive processes allocate and coordinate attentional resources in task performance^26,27^, with control over attention playing a role in governing intruding thoughts during comprehension^28^. Neuroimaging studies have revealed that regions associated with executive control, including the left dorsolateral prefrontal cortex, show greater activation in better readers^29,30^. Finally, reading comprehension involves conceptual processing that goes beyond single word decoding to include text-level and often discourse-level inference – such as integration of text-based information with episodic memory and real-world knowledge^31,32^, forming what is often referred to as the situation model^33^. Converging evidence has revealed a role for medial prefrontal cortex in inference processes while reading passages^34–36^. Deep comprehension of texts is also associated with successful recognition and better memory for the information they contain^31,37^.

Contemporary accounts of off-task thought also suggest that this experience emerges from the interaction of multiple component processes^38^. At the core of the off-task state is a shift in focus away from the external task towards self-generated mental content, known as decoupling^4,38^. This process is assumed to reflect a disengagement of neural processing from external input that occurs because the attentional focus has changed, as evidenced by reductions in evoked responses to externally-presented inputs during off-task thought^39–41^. While the process of decoupling is argued to support our ability to generate thoughts unrelated to the external world, control processes are thought to be important in maintaining the integrity of the desired pattern of thoughts^38,42^. Individuals with higher executive control tend to be better at maintaining attention to task-relevant information in attentional tasks^43^ and whilst reading for comprehension^28^. Recent research suggests task focus may be implemented by dorsal and ventral attention systems^43,44^. The ventral attention network – in particular the left dorsolateral prefrontal cortex – plays a role in the prioritisation of cognition appropriate to the context in sustained attention tasks, while the dorsal attention network reflects a focus on tasks regardless of the context. Yet a different picture emerges from an examination of the neural correlates of on- and off-task thought during reading. In a prior study, we found that individuals who maintain a better focus on what is being read, when tested outside the scanner, show greater within-network connectivity between regions of the default mode network^45^, thought to be important for retrieval from semantic and episodic memory^46^. One possibility is that this difference reflects the conceptual and episodic representations that are needed for reading, which also contribute to the mental content that is generated during off-task thought^47^.

In the current study, we used functional neuroimaging to revisit the specific neurocognitive mechanisms that contribute to individual variation in off-task thought during reading. We recruited a cohort of 69 participants and asked them to read an expository non-fiction text outside the scanner whilst keeping track of the amount of mind-wandering they experienced^3^. Immediately afterwards, participants completed a set of questions assessing their memory for information from the text. These questions required the recall of information directly presented in the text, and in some cases, the integration of additional semantic information to understand the text^31,48^. Although people’s eyes are still scanning the text during periods of mind-wandering, we expected that during these off-task states, the orthographic input would receive less attention and subsequent semantic processing^49^, resulting in poorer memory for the text.

In Experiment 1, we employed a sentence-reading task in the scanner, in which participants were asked to passively view meaningful sentences or nonwords, presented on an item-by-item basis. This task was expected to activate the visual-to-semantic pathway, implicated in reading comprehension^11,50^ and was used to characterise receptive language function in individual participants^51,52^. Previous studies examining the neural correlates of reading have revealed greater involvement of a temporo-frontal semantic network for meaningful versus meaningless texts, including greater activation in superior temporal gyrus/sulcus, middle temporal gyrus, angular gyrus and inferior frontal gyrus^53,54^. We therefore predicted that both meaningful and meaningless orthographic inputs would elicit a response within the ventral visual pathway, with additional activation in regions within a temporo-frontal semantic network when contrasting words with nonwords. Accordingly, individual differences in memory for texts might reflect differences in conceptual retrieval, related to the contrast *words* > *nonwords*, and/or the extent to which people focus on reading instead of mind-wandering even when the orthographic input is meaningless, using the contrast of *nonwords* > *baseline*. We examined whether differences in BOLD activation during the sentence-reading tasks would predict variation in patterns of experience during reading or variation in text-based memory (both measured outside of the scanner). In Experiment 2, we collected a resting-state scan in the same individuals, allowing us to understand whether the connectivity patterns of regions identified in the first experiment varied with either mind-wandering during reading, or subsequent text-based memory. To foreshadow our results, Experiment 1 identified a region of middle temporal gyrus within the default mode network (DMN) that had reduced neural activity in response to orthographic input in individuals who had poorer memory for texts. Experiment 2 established that this region of middle temporal gyrus had stronger connectivity with regions of the medial prefrontal cortex for individuals with good text-based memory, and had weaker connectivity with regions of medial occipital cortex for individuals showing more off-task thought.

## Materials and Methods

### Participants

Ethical approval was obtained from the Research Ethics Committees of the Department of Psychology and York Neuroimaging Centre, University of York. And all research was performed in accordance with the relevant guidelines/regulations. Sixty-nine undergraduate or postgraduate students were recruited for this study (age range 18–31, mean age = 19.87 ± 2.33, 26 males). All were right-handed native English speakers, and had normal or corrected-to-normal vision. None of them had any history of neurological impairment, diagnosis of learning difficulty or psychiatric illness. All provided written informed consent prior to taking part and received a monetary reward for their participation.

### Behavioural assessment

*Off-task frequency and text-based memory*. Following an MRI scan (details below), participants were asked to complete a battery of behavioural assessments examining their reading and off-task thought. In order to create a naturalistic reading experience, we presented the text in a printed booklet. The text was selected and shortened from Bill Bryson’s “A Short History of Nearly Everything”^45^, in font size 14, 1.5 line spacing. The passage was about the topic of geology (word count = 1050). During reading, the participants were required to note down any moments when they noticed they had stopped paying attention to the meaning of the text, by circling the word they had reached at this point. A detailed instruction booklet was used to guide the participants through the experiment. After they finished reading, they were asked to answer 17 open-ended questions, without being able to refer back to the text^45^. For some questions, the answers were explicitly presented in the text (i.e., *How high was the annual membership fee for the geological society?* or *How old did Holmes estimate the earth to be?* or *What kind of interim business did Holmes run to be able to support his family?*), while others required text memory but also required participants to retrieve and integrate semantic information to support their understanding of the paragraph and to answer the question (i.e., *Why does the author compliment about Holmes first estimation of the age of the earth as ‘quite an achievement’? or Which circumstance was responsible for delaying Holmes’ work?*). The questions did not require discourse-level comprehension.

The answers to the questions were scored for accuracy by two experimenters. Responses were given a score of 1 if they contained key information, and otherwise a score of 0. The two scorers produced very similar ratings (*r* = 0.92, *p* < 0.001). The prior knowledge of geology was also assessed by two questions: (1) Have you previously studied Geology, and if so for how many years? (2) Please indicate how much you already knew about the content of the Geology text prior to reading the text based on a 0 to 10 Likert scale. The participants stated that they were unfamiliar with the content of the text (*M* ± *SD* = 1.47 ± 1.75) and had almost no Geology study during their education (*M* ± *SD* = 0.06 ± 0.29 year). There was also no correlation between these ratings with either reading assessment or off-task experiences (*ps* > 0.1). The experiment took approximately 30 (±5) minutes.

*Off-task experiences*. A self-report measurement, the New-York Cognition Questionnaire (NYC-Q), was also used to assess off-task behaviour during the reading task. The first section contained 22 questions about the content of thoughts (e.g., *I thought about personal worries*), rated on a scale of 1 (*Completely did not describe my thoughts*) to 9 (*Completely did describe my thoughts*). The second section contained 8 questions about the form of these thoughts (e.g., *whilst I was reading my thoughts were in the form of images*), rated on a scale of 1 (*Completely did not characterize my experience*) to 9 (*Completely did characterize my experience*)^3,55^. In the current study, we limited our analysis to the 22 questions relating to the content of off-task thought. We calculated an overall average for each participant, which is thought to reflect how much each individual was thinking off-task thoughts. In this way, we assessed both off-task frequency (i.e., the number of moments when attention was not directed towards the reading task) and the content of these experiences.

Prior to data analysis, all variables were *z*-transformed and outliers more than 2.5 standard deviations above or below the mean were identified. Using this criterion, there was one outlier for text-based memory, three outliers for off-task frequency and no outliers in the content of off-task thought questionnaire. These outlying values were imputed with the cut-off value (i.e., +/−2.5 standard deviations above or below the mean). No participant was removed from the data analysis as a result of this process.

### Neuroimaging data acquisition

Structural and functional data were acquired using a 3T GE HDx Excite Magnetic Resonance Imaging (MRI) scanner utilizing an eight-channel phased array head coil at the York Neuroimaging Centre, University of York. Structural MRI acquisition in all participants was based on a T1-weighted 3D fast spoiled gradient echo sequence (repetition time (TR) = 7.8 s, echo time (TE) = minimum full, flip angle = 20°, matrix size = 256 × 256, 176 slices, voxel size = 1.13 mm × 1.13 mm × 1 mm).

The sentence-reading task in Experiment 1 used single-shot 2D gradient-echo-planar imaging (TR = 3 s, TE = minimum full, flip angle = 90°, matrix size = 64 × 64, 60 slices, voxel size = 3 mm × 3 mm × 3 mm, 80 volumes). The participants passively viewed meaningful sentences (e.g., her + secrets + were + written + in + her + diary) and meaningless sequences of nonwords (e.g., crark + dof + toin + mesk + int + lisal + glod + flid), item-by-item. In total, there were 10 meaningful sentences, taken from Rodd, *et al*.^56^, and 10 nonword lists, matched for both word length and number of syllables. Word and nonword sets were each presented in two blocks in a pseudo-random order (i.e., a total of 4 blocks). A task instruction (e.g., Meaningful) was used to indicate the transition between different conditions. Each sequence ended with a red fixation lasting 4000–6000 ms. Each word or nonword was presented for 600 ms, followed by a 250 ms fixation before the next item was presented. A fluid-attenuated inversion-recovery (FLAIR) scan with the same orientation as the functional scans was collected to improve co-registration between subject-specific structural and functional scans.

A 9-minute resting-state fMRI scan was used in Experiment 2, recorded using single-shot 2D gradient-echo-planar imaging (TR = 3 s, TE = minimum full, flip angle = 90°, matrix size = 64 × 64, 60 slices, voxel size = 3 mm × 3 mm × 3 mm, 180 volumes). During resting-state scanning, the participants were instructed to focus on a fixation cross with their eyes open and to keep as still as possible, without thinking about anything in particular. Neuroimaging data for Experiments 1 and 2 were collected in the same session, with the resting-state sequence presented first, so that measures of intrinsic connectivity could not be influenced by the words and nonwords that were presented in the sentence-reading task. There was a break of a few minutes between these scans which allowed us to remind participants of the task requirements and set up the scanning.

### Neuroimaging data pre-processing

All functional and structural data were pre-processed using a standard pipeline and analysed via the FMRIB Software Library (FSL version 6.0, www.fmrib.ox.ac.uk/fsl). Individual FLAIR and T1-weighted structural brain images were extracted using FSL’s Brain Extraction Tool (BET). Structural images were linearly registered to the MNI152 template using FMRIB’s Linear Image Registration Tool (FLIRT). The sentence-reading functional neuroimaging data were pre-processed and analysed by using FSL’s FMRI Expert Analysis Tool (FEAT). A standard pre-processing pipeline was applied, including motion correction via MCFLIRT, slice-timing correction using Fourier space time-series phase-shifting, and spatial smoothing using a Gaussian kernel of FWHM 6 mm. In addition, for the task-based fMRI data in Experiment 1, high-pass temporal filtering (sigma = 100 s) was applied in order to remove temporal signal drift. For the resting-state fMRI data in Experiment 2, both high-pass (sigma = 200 s) and low-pass temporal filtering (sigma = 2.8 s) were applied, in order to constrain analyses to low-frequency fluctuations.

### Neuroimaging analysis

#### Task-based fMRI analysis (Experiment 1)

This analysis identified sites in which activation during the sentence reading task was modulated by individual differences in text-based memory, off-task frequency or the content of off-task thought (i.e., NYC-Q), measured outside the scanner. In the first-level analysis of the sentence reading task performed in the scanner, we identified voxels responding to (i) meaning and (ii) orthographic inputs devoid of meaning, through the contrasts of *Meaningful* > *Baseline*, *Meaningless* > *Baseline*, *Meaningful* > *Meaningless*, plus the reverse, for each participant. In the higher-level analysis at the group level, z-transformed behavioural data for text-based memory, off-task frequency and NYC-Q were added as explanatory variables, using FMRIB’s Local Analysis of Mixed Effects (FLAME1), with automatic outlier de-weighting^57^. A 50% probabilistic grey-matter mask was applied. Clusters were thresholded using Gaussian random-field theory, with a cluster-forming threshold of *z* = 2.6 and a familywise-error-corrected significance level of *p* = 0.05.

#### Resting-state fMRI analysis (Experiment 2)

We next considered whether the intrinsic connectivity of regions identified in Experiment 1 predicted text-based memory and off-task thought. A cluster which showed a stronger response to orthographic input in people with good text-based memory overlapped with (i) the default mode network (DMN), which is implicated in both reading comprehension and spontaneous thought^46,47,58^, (ii) the adjacent frontoparietal network (FPN), which plays a central role in cognitive control^59^, as well as (iii) the dorsal attention network (DAN), which supports externally-directed attention^60^. We therefore masked the results of Experiment 1 by these DMN, FPN and DAN networks, defined by a parcellation of 1000 resting-state scans^61^, obtained from Freesurfer (https://surfer.nmr.mgh.harvard.edu/fswiki/CorticalParcellation_Yeo2011). This identified a region of middle temporal gyrus (MTG) within DMN, and a region of inferior temporal gyrus (ITG) in the FPN, as well as another region of inferior temporal region (ITG)/lateral occipital cortex (LOC) in the DAN. These regions were taken as seeds in a subsequent analysis of intrinsic connectivity.

We extracted the time series from the seeds and used this data as explanatory variables in whole-brain connectivity analyses at the single-subject level. Sixty-four participants were included in this analysis (five participants without intrinsic connectivity data were excluded). These functional connectivity maps were then related to individual differences in behaviour using a multiple regression model, in which z-transformed scores for text-based memory, off-task frequency and NYC-Q were added as explanatory variables. In order to control for the spurious correlations that might emerge from movement, we included two canonical components, group mean and mean framewise displacement (FD)^62^, as nuisance covariates in the model. Automatic outlier de-weighting was used and a 50% probabilistic grey-matter mask was applied. Clusters were thresholded using Gaussian random-field theory, with a cluster-forming threshold of *z* = 2.6 and a familywise-error-corrected significance level of *p* = 0.05. We also applied Bonferroni correction to account for the fact that we included three models (ITG within FPN, ITG/LOC within DAN, and MTG within DMN) and used two-tailed tests (in which behaviour could relate to both stronger and weaker connectivity). Consequently, the *p*-value accepted as significant was *p* < 0.0083.

## Results

### Behavioural results

**Figure 1:**
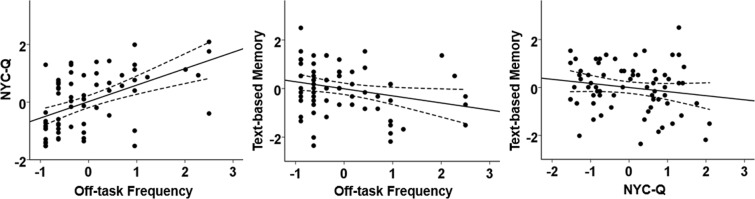
Behavioural results. The scatterplots present the correlations between off-task frequency, NYC-Q (i.e., the content of off-task thought), and text-based memory. The error lines on the scatterplots indicate the 95% confidence estimates of the mean. Each point on the scatterplot describes a participant.

The behavioural results are summarized in Fig. 1. Pearson’s correlation analysis revealed that the online (i.e. off-task frequency; *M* ± *SD* = 3.38 ± 3.79) and retrospective measures of off-task experience (i.e. NYC-Q; *M* ± *SD* = 3.09 ± 1.37) were positively correlated (*r* = 0.51, *p* < 0.001). There was also a significant negative correlation between off-task frequency and text-based memory scores (*M* ± *SD* = 7.96 ± 2.95; *r* = −0.26, *p* = 0.029), suggesting that frequent off-task thought interferes with reading, in line with previous findings^1,3^. However, the retrospective mind-wandering measure was not significantly associated with text-based memory (*r* = −0.17, *p* = 0.16).

### Experiment 1: sentence-reading task

**Figure 2:**
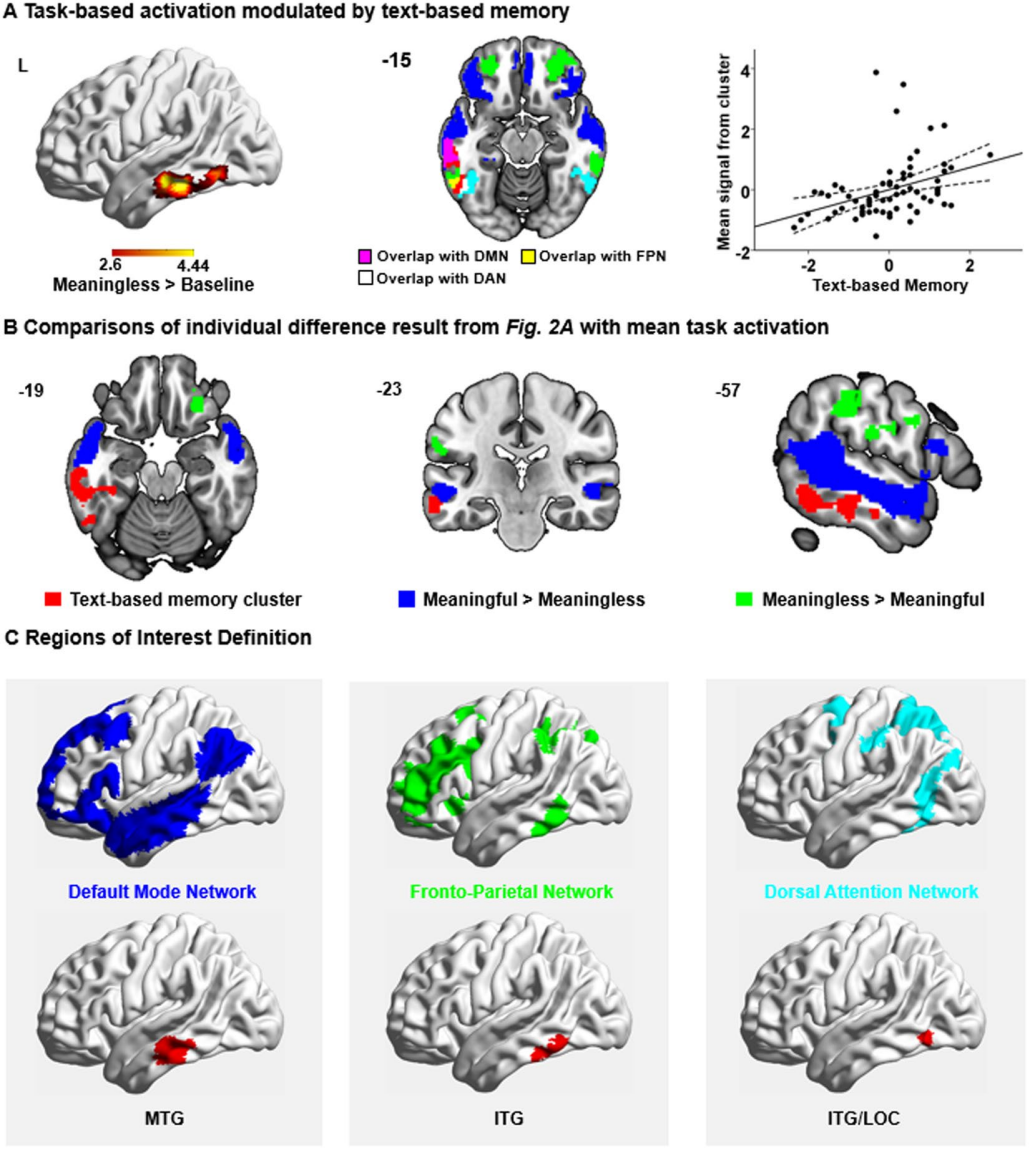
(**A**) Regions modulated by text-based memory in task-based fMRI. Participants were asked to passively read meaningful sentences and meaningless sequences of non-words. The image shows voxels that were more active for good readers for the contrast of meaningless nonwords over baseline (*z* > 2.6; *p* < 0.05; in red). This cluster overlaps with three large-scale brain networks: default mode network (DMN; in blue), frontoparietal network (FPN; in green), and dorsal attention network (DAN; in cyan). These networks were defined by Yeo, *et al*.^61^, in a 7-network parcellation of whole-brain functional connectivity for 1000 brains. The network map is fully saturated to emphasize the regions of overlap (in pink, yellow, and white). The number in the top left of the overlap map indicates the coordinate value of the corresponding plane. The scatterplot presents the correlation between the mean signal extracted from the significant cluster and text-based memory. The error lines on the scatterplot indicate the 95% confidence estimates of the mean. Each point describes an individual participant. L = Left hemisphere. (**B**) Comparisons of individual difference result from Fig. 2A with mean task activation. The brain region in which the response to orthographic input was modulated by text-based memory (red) with the activated regions in the contrasts of *Meaningful* > *Meaningless* (blue) and *Meaningless* > *Meaningful* (green). These maps are fully saturated. All maps are thresholded at *z* > 2.6 (*p* < 0.05). Numbers at the top left of each panel indicate the coordinate value of the corresponding plane. (**C**) Definition of task-based ROIs. The seeds used for resting-state functional connectivity in Experiment 2 were defined as the overlaps between the region in which task-based activation to meaningless orthographic input was modulated by text-based memory and three large-scale brain networks: DMN, FPN, and DAN. This gave rise to three ROIs: middle temporal gyrus (MTG) in DMN, a region of inferior temporal gyrus (ITG) in FPN, as well as a region of ITG/lateral occipital cortex (LOC) in DAN.

A whole-brain analysis indicated that activation in middle/inferior temporal gyrus and temporal fusiform cortex in the contrast of *Meaningless* > *Baseline* was modulated by individual differences in text-based memory, measured outside the scanner (*p* = 0.0111; see Fig. 2A). To understand the nature of this relationship, we plotted the relationship between mean % signal change in this region and text-based memory across individuals. People with better text-based memory showed a stronger response to orthographic input, even when this was not meaningful.

We did not find any clusters for the *Meaningful* > *Meaningless* contrast that varied with individual differences in either text-based memory or off-task thought. To identify whether the association with text-based memory in *Meaningless* > *Baseline* showed a similar pattern to *Meaningful* > *Baseline*, we used the cluster identified in the contrast of *Meaningless* > *Baseline* as a mask to extract the mean % signal change in the contrast of *Meaningful* > *Baseline* for each individual, then performed Bayesian Pearson Correlation Inference analysis to quantify the relationship between the text-based memory score and activation in the *Meaningful* > *Baseline* contrast. The estimated Pearson correlation coefficient was 0.30, *p* = 0.012, and the corresponding Bayes Factor was 0.46. This score is a natural ratio comparing the likelihood of no correlation with the likelihood of a correlation between text-based memory and brain activation for the *Meaningful* > *Baseline* contrast. Since the Bayes Factor score was less than 1, this suggests that there is ‘moderate evidence’ that the association is present in both conditions. Consequently, the effect of text-based memory on the BOLD response is most likely to reflect a generally greater response to orthographic input in participants with better text-based memory.

We compared the location of this temporal lobe cluster that reflected a stronger BOLD response to orthographic inputs in participants with good text-based memory (shown in red in Fig. 2B) with the main effect of meaning across the group in Experiment 1. The location of this temporal lobe, which showed an effect of individual differences, was adjacent on the cortical surface to the mean task activation in the contrast of *Meaningful* > *Meaningless* (shown in blue in Fig. 2B). This temporal lobe activation is implicated in visual-to-semantic processes in contemporary accounts that propose graded abstraction from unimodal visual to heteromodal conceptual representations within the temporal lobe^5^.

The cluster that showed a stronger response to orthographic input in people with text-based memory was located at the intersection of DMN, FPN and DAN (see Fig. 2A). We calculated the percentage of voxels within this cluster that fell within the large-scale networks defined by Yeo, *et al*.^61^. Of these voxels, 48.1% were within the DMN (overlap in pink), which is implicated in both reading comprehension and spontaneous thoughts^46,47,58^; 17.4% fell within the adjacent FPN (overlap in yellow), which plays a central role in cognitive control^59^; and 24.8% fell within the DAN (overlap in white), which supports externally directed attention^60^. There were smaller overlaps with limbic and visual networks, 8% and 1.8% respectively, which we do not discuss further.

### Experiment 2: Resting-state functional connectivity

Since the region that showed greater responsiveness to orthographic input in people with good text-based memory fell at the intersection of three large-scale networks (DMN, FPN, DAN), we conducted a second experiment to understand whether the organisation of one of these large-scale networks also relates to individual differences in off-task thinking and reading. We therefore identified voxels that showed greater activation to orthographic input for people with good text-based memory in Experiment 1, which also fell within DMN, FPN, and DAN – implicated respectively in memory, control, and attention processes according to a commonly used whole-brain parcellation^61^. We used these DMN, FPN, and DAN regions as seeds for an analysis of intrinsic connectivity in Experiment 2 (see Fig. 2C).

**Figure 3:**
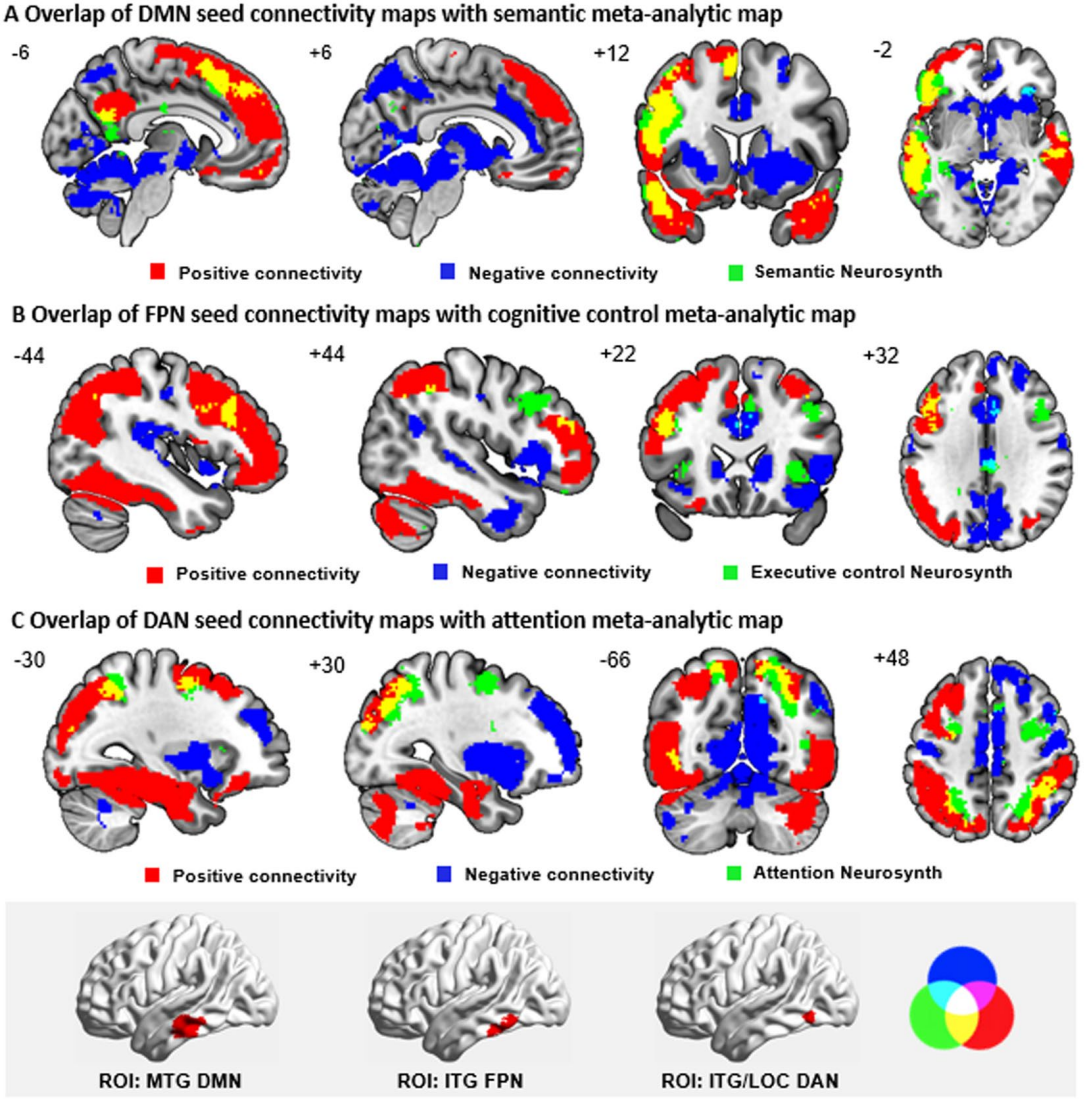
(**A**) Overlap of DMN seed connectivity maps with semantic meta-analytic map. The group-level patterns of relatively high (in red) and low (in blue) functional connectivity from the DMN seed in MTG during resting-state fMRI (cluster correction, *z* > 2.6, *p* < 0.05), and the overlap of these positive and negative networks with a semantic meta-analytic map (regions in green) derived from Neurosynth (using ‘semantic’ as a search term). (**B**) Overlap of FPN seed connectivity maps with cognitive control meta-analytic map. The group-level patterns of relatively high (in red) and low (in blue) functional connectivity from the FPN seed in ITG during resting-state fMRI (cluster correction, *z* > 2.6, *p* < 0.05), and the overlap of these positive and negative networks with a cognitive control meta-analytic map (regions in green) derived from Neurosynth (using ‘cognitive control’ as a search term). (**C**) Overlap of DAN seed connectivity maps with attention meta-analytic map. The group-level patterns of relatively high (in red) and low (in blue) functional connectivity from the DAN seed in ITG/LOC during resting-state fMRI (cluster correction, *z* > 2.6, *p* < 0.05), and the overlap of these positive and negative networks with attention meta-analytic map (regions in green) derived from Neurosynth (using ‘attention’ as a search term). These maps are fully saturated to emphasize the regions of overlap. Numbers at the top left of each panel indicates the coordinate value of the corresponding plane. The bottom panel highlighted in grey shows the seed regions and the overlapping circles that indicate the colour of overlap regions. MTG = middle temporal gyrus. ITG = inferior temporal gyrus. LOC = lateral occipital cortex.

Group-level intrinsic connectivity maps for the DMN, FPN, and DAN seed regions (i.e., irrespective of performance) are presented in Fig. 3. To understand how the regions of positive and negative connectivity from these seed regions respectively correspond to the networks implicated in semantic, cognitive control, and attention processing, we compared these spatial maps to the meta-analytic maps generated for the terms semantic, cognitive control, and attention using Neurosynth^63^. This revealed that regions of relatively high connectivity from DMN, FPN, and DAN seed regions respectively (shown in red in Fig. 3) largely overlapped with regions important for these aspects of cognition according to task-based fMRI (shown in green, with the overlap in yellow).

### Relationship to individual differences

We explored whether individual differences in performance on the factual questions about the text and off-task thought were associated with variation in patterns of intrinsic connectivity from these seeds. We generated functional connectivity maps for each region, for each individual, and then analysed these spatial maps using a series of multiple regression analyses that included individual scores in off-task thought (i.e., off-task frequency and NYC-Q) and text-based memory as explanatory variables. There were no significant differences in the connectivity of the FPN seed region in ITG that related to either off-task thought or text-based memory, so this site is not discussed further. There were some effects for the DAN seed in ITG/LOC but these failed to survive Bonferroni correction for the number of seeds and the two-tailed nature of our tests (see Section 3.3.3.2.).

#### Performance on factual questions related to the text

**Figure 4:**
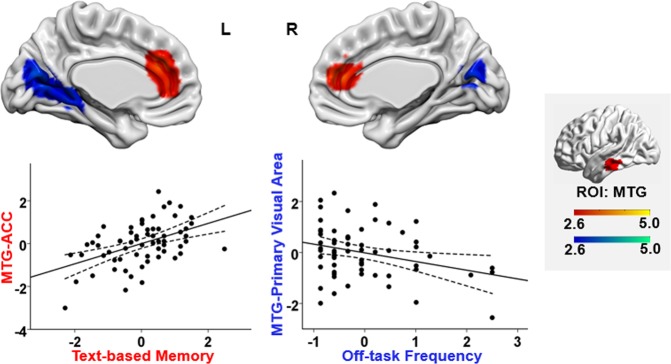
Functional connectivity of MTG within DMN linked to off-task frequency and text-based memory. The regions in red showed stronger connectivity to MTG for participants with good text-based memory, while the regions in blue showed weaker connectivity with MTG for participants with more frequent off-task thought. All maps are cluster corrected at a threshold of *z* > 2.6 (*p* < 0.05). The scatterplots present the correlation between behaviour (text-based memory or off-task frequency) and the average correlation with the MTG seed and the relevant cluster (beta values). The error lines on the scatterplot indicate the 95% confidence estimates of the mean. Each point describes an individual participant. The right-hand panel highlighted in grey shows the seed region and the colour bars. MTG = middle temporal gyrus; ACC = anterior cingulate cortex.

We found that MTG connectivity was related to memory for the content of the text. Participants with better text-based memory scores showed stronger connectivity between the MTG DMN seed region and anterior cingulate cortex (cingulate gyrus and paracingulate gyrus; uncorrected *p* = 0.006). This cluster is illustrated in Fig. 4. Of the voxels within the anterior cingulate cluster that fell within the large-scale networks defined by Yeo, *et al*.^61^, 88.3% were within DMN, 11.3% fell within FPN, and 0.4% fell within ventral attention network. These findings show that connectivity between different nodes of DMN is linked to better text-based memory (not poorer performance, as a task-negative theory of DMN might predict).

#### Off-task Frequency

We also found that increasing off-task frequency was associated with weaker connectivity between MTG in DMN and visual cortex (intracalcarine cortex, precuneus cortex, and lingual gyrus; uncorrected *p* = 0.006). This effect is presented in Fig. 4. Of the voxels in this cluster that fell within one of the large-scale networks defined by Yeo, *et al*.^61^, 100% were within the visual network. Consequently, participants with stronger intrinsic connectivity at rest between DMN and visual cortex were less likely to engage in off-task thinking while reading.

#### Additional effects

There were several additional effects that were significant at the whole-brain level that did not survive Bonferroni correction for the number of models and the two-tailed nature of our tests (e.g., *p* > 0.0083). When considering these results, it is important to note that these may reflect Type II errors and should be considered accordingly.

**Figure 5:**
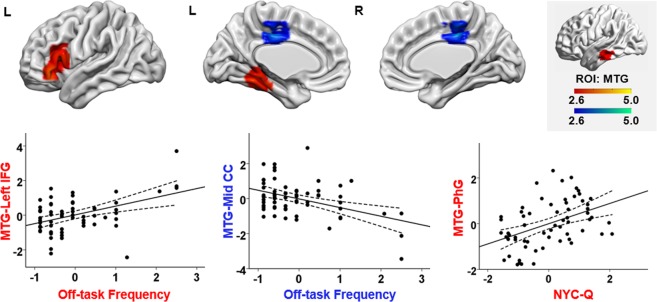
Additional effects of functional connectivity seeding from MTG DMN seed region. The regions in warm colour indicate regions displaying connectivity with MTG were stronger for the participants with frequent off-task thought or higher NYC-Q scores. And the regions in cold colour indicate regions displaying connectivity with MTG were weaker for the participants with frequent off-task thought. All maps are cluster corrected at a threshold of *z* > 2.6 (*p* < 0.05). The scatterplots present the relationship between the average correlation with MTG (beta values) in each region and off-task frequency, as well as NYC-Q scores. The error lines on the scatterplots indicate the 95% confidence estimates of the mean. Each point describes each participant. The upper right panel highlighted in grey shows the seed region and the colour bar. IFG = inferior frontal gyrus; Mid CC = middle cingulate cortex; PhG = parahippocampal gyrus; MTG = middle temporal gyrus.
(ii) Increasing off-task thought was associated with greater connectivity between left inferior frontal gyrus and the DMN portion of MTG (uncorrected *p* = 0.044). Of the voxels within left inferior frontal gyrus that fell within the large-scale networks defined by Yeo, *et al*.^61^, 69.5% were within the default mode network, 16.9% fell within the frontoparietal network, and 13.6% fell within the ventral attention network.
(iii) Higher NYC-Q scores were linked to greater connectivity between MTG and parahippocampal gyrus as well as temporal fusiform cortex (uncorrected *p* = 0.028). Of the voxels within parahippocampal gyrus that fell within the large-scale networks defined by Yeo, *et al*.^61^, 96.6% were within the visual network and 3.4% fell within the default mode network.

Seeding from MTG DMN region:

(i) With increasing off-task thought, a mid-cingulate cortex region showed greater disconnection with MTG (uncorrected *p* = 0.022; see Fig. 5). Of the voxels in this cluster that fell within the large-scale networks defined by Yeo, *et al*.^61^, 38.1% were within the somatomotor network, 47.6% fell within the default mode network, and 7.6% and 6.7% fell within the ventral attention and frontoparietal network, respectively. This pattern in some ways resembles decoupling from the visual cortex, at a lower statistical threshold.

**Figure 6:**
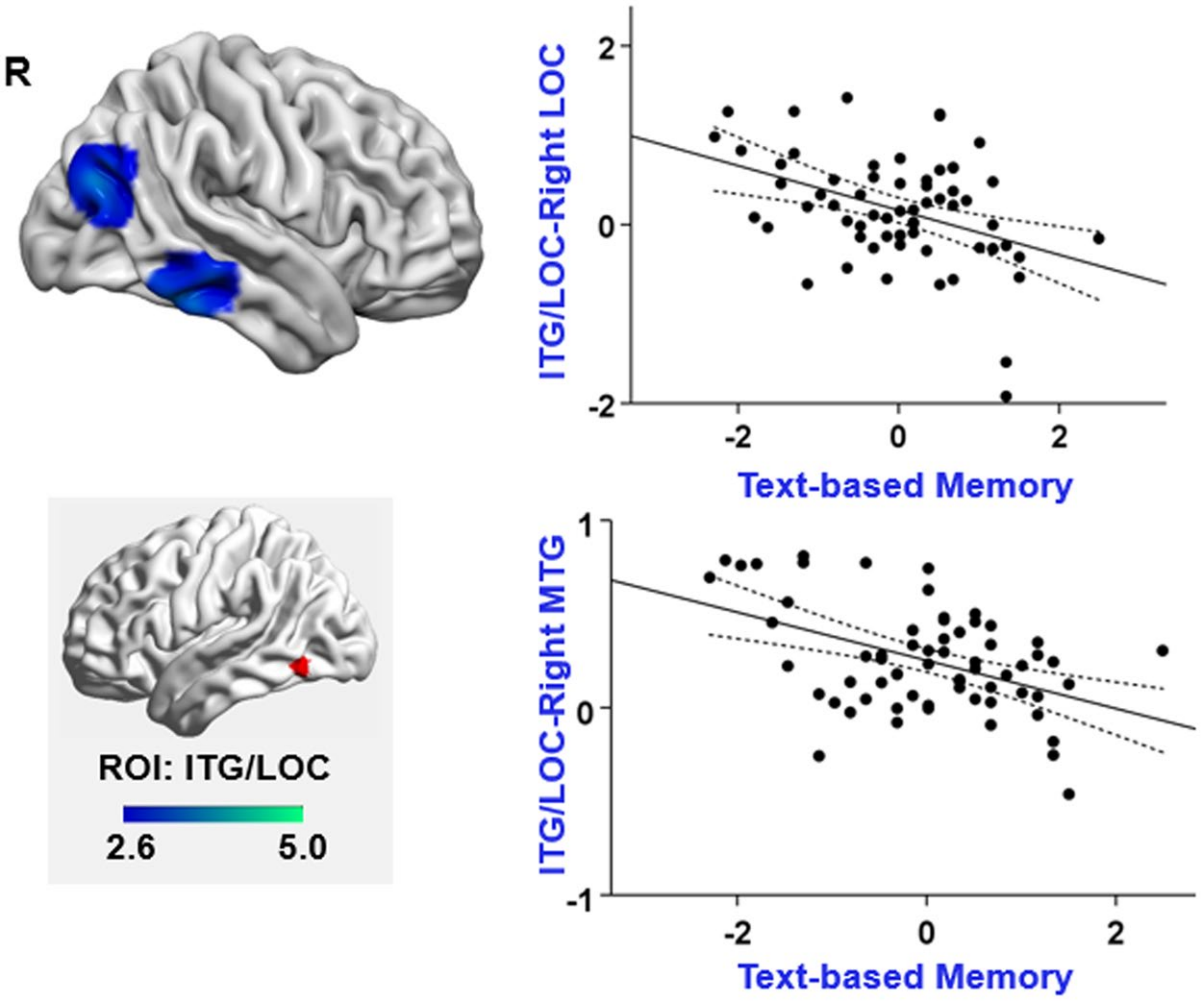
Additional effects of functional connectivity seeding from ITG/LOC DAN seed region. The regions in cold colours indicate regions displaying weaker connectivity with ITG for participants with good text-based memory. All maps are cluster corrected at a threshold of *z* > 2.6 (*p* < 0.05). The scatterplots present the relationship between the average correlation with ITG (beta values) in each region and text-based memory. The error lines on the scatterplots indicate the 95% confidence estimates of the mean. Each point describes each participant. The lower left panel highlighted in grey shows the seed region and the colour bar. R = right hemisphere; Right LOC = right lateral occipital cortex; Right MTG = right middle temporal gyrus; ITG = inferior temporal gyrus.

Seeding from ITG/LOC DAN region: Analysis of the functional connectivity of DAN in ITG/LOC showed that poor text-based memory was linked to greater connectivity between the left ITG and (i) the right lateral occipital cortex (uncorrected *p* = 0.041; see Fig. 6) as well as (ii) the right inferior and middle temporal gyrus (uncorrected *p* = 0.015). For the voxels within the right lateral occipital cortex that fell within the large-scale networks defined by Yeo, *et al*.^61^, 56.8% fell within the dorsal attention network, 28.4% were within visual network, 5.8% fell within default mode network, and 2.3% fell within the frontoparietal network. For the voxels within the right inferior/middle temporal gyrus that also fell within the large-scale networks defined by Yeo, *et al*.^61^, 69.8% fell within the frontoparietal network, 29.3% were within dorsal attention network, and 0.8% fell within the default mode network.

### Summary of results

Our study set out to examine the neural mechanisms that contribute to reading performance, assessed using factual questions answered from memory about the content of a text, and periods of off-task thinking during reading. Using a data-driven approach, Experiment 1 identified regions within the middle temporal gyrus that activated more for people with better text-based memory in the sentence-reading task. This region fell at the intersection of three large-scale networks – the DAN, FPN and DMN. In Experiment 2, we separated the MTG cluster into sub-regions that corresponded to each of these networks and used them in an analysis of resting-state connectivity to establish whether the intrinsic architecture of any of these regions was predictive of variation in either patterns of off-task thought or text-based memory. We found no evidence that variation in either memory for the text or off-task thought was linked to the connectivity of the FPN region. Weak evidence emerged for a role of the DAN in reading: weaker connectivity with regions of both the right angular gyrus/lateral occipital cortex and right middle temporal gyrus was linked to better text-based memory, although this result did not survive correction for the number of seeds and the two-tailed nature of our tests. The strongest evidence was observed for the DMN since the MTG sub-region showed patterns of connectivity that were linked to both reading and off-task thinking. The most robust of these effects were (i) stronger connectivity with a medial prefrontal region, also in DMN, that was associated with better text-based memory, and (ii) weaker connectivity with visual cortex, linked to greater frequency of off-task thought. Together these results provide converging support for the role of multiple large-scale systems in the ability to read for meaning, and specific support for the importance of the DMN in both memory for texts, as well as for the occurrence of off-task thought during reading.

## General Discussion

We show how a region of the lateral temporal cortex within the default mode network (DMN) is implicated in both on-task processing (i.e. text processing) and off-task mental states (such as periods of off-task thought). In Experiment 1, which employed a sentence-reading task in the scanner, we found people who were better able to answer questions about a text that they had read previously showed a greater BOLD response to orthographic inputs in middle temporal gyrus (MTG) within DMN, and a region of inferior temporal gyrus (ITG) in FPN, as well as another region of ITG/lateral occipital cortex in DAN. In Experiment 2, we explored individual differences in the intrinsic connectivity of these sites. For individuals with more frequent off-task thought, MTG within DMN showed weaker connectivity with visual cortex, suggesting that perceptual decoupling may promote off-task thought. In contrast, for individuals with better text-based memory, MTG showed greater connectivity with anterior cingulate cortex, also in the DMN. These findings show that DMN regions in lateral temporal cortex have patterns of connectivity that uniquely support both reading, and states that are detrimental to making sense of what one is reading (i.e. off-task thought).

Although our results show relatively clear evidence for the involvement of the DMN in both reading and off-task thought, our study failed to find robust evidence supporting a role of the DAN or the FPN. No whole-brain results were observed for the FPN. For the DAN, better text-based memory was associated with weaker correlation between regions of the right temporal and parietal cortex that fell largely within the FPN and DAN. Although it may seem paradoxical that higher connectivity within the DAN is linked to poorer reading performance, our prior studies using a sustained attention task found lower connectivity within this system (and in particular to the same LOC cluster) was linked to a greater tendency for attention to remain coupled to the task^43^. Moving forward, our studies suggest that it will be important to assess the neural basis of different features of experience across multiple task contexts.

Our results add to a growing body of evidence that DMN plays a complex role in reading. Our prior study found that DMN connectivity to different regions was linked to both better and poorer comprehension. The current study shows that the MTG region may be important in the capacity of the DMN to contribute to apparently opposing mental states. Our study suggests that rather than different temporal lobe regions supporting on-task and off-task semantic retrieval (for example, regions falling within FPN and DMN respectively), diverse patterns of connectivity from the same DMN region can underpin both off-task thought and reading. It has already been observed that semantic regions within temporal cortex have a pattern of connectivity to both DMN core and visual cortex^19,64,65^ – our results suggest that both of these connections are important for good text-based memory. Our results are also broadly consistent with the recent observation that while DMN often shows a response to nonwords over words (reflecting off-task processing), these DMN regions also support semantic processes engaged in reading^66^.

Functionally, MTG is implicated in heteromodal aspects of cognition as the inputs along auditory and visual processing streams maximally converge here^5,19,64,67^. MTG responds more strongly to memory-based and meaning-based decisions, consistent with the location of this cluster at the anterior end of the ventral visual stream within the DMN^68^. In line with these studies, the anterior and middle temporal lobe have been identified as important for text comprehension^9,69,70^. Nevertheless, activation within MTG is insufficient for comprehension – our results suggest this region also needs to be strongly activated by visual inputs and to interact with other regions of DMN implicated in comprehension.

Finally, our study also provides evidence that poor reading is linked to inattention or perceptual disengagement^38,71^. First, we found that poor memory for texts was linked to lower levels of activity in MTG in response to orthographic inputs. Since this response was identified in the meaningless condition, it is likely to be a consequence of how perceptual input rather than meaning is processed. Second, we found that the aspect of this region that fell within the DMN was less coupled to primary visual cortex for individuals who were frequently off-task while reading. Event-related potentials evoked by sensory inputs are reduced in magnitude during episodes of off-task thought, relative to on-task periods^40^. The posterior core of the DMN which supports heteromodal integration^72^ also contributes to different types of spontaneous thought^47^. The role of MTG in processing texts and in off-task thought may be similar: perceptual decoupling of MTG from visual cortex may allow this region to support off-task thought that is unrelated to the immediate external environment. This pattern is generally consistent with the cascade model of inattention^71^, which argues that off-task states during reading partly reflect reductions in perceptual processing. This leads to cascading consequences affecting both comprehension and memory for texts^1^.

While DMN regions support off-task states that impair text processing, connectivity within DMN predicted good memory for texts. In people who were able to answer more factual questions about what they had read, MTG coupled more with anterior cingulate cortex, and both regions fell largely within DMN as defined by Yeo, *et al*.^61^. Both of these regions are ‘hubs’ that integrate diverse elements of cognition^64,67,73,74^. Anterior cingulate cortex shows graded connections at rest with both sensory and motor cortices, as well as with memory/DMN regions^75^. Extrapolating from our results, individuals who had the most efficient reading experience (i.e. who experienced less off-task thought and answered more questions about the text) would show a combination of strong within-DMN connectivity and less decoupling with the visual system. Although there are likely to be functional subdivisions within DMN, findings from our study directly concerned with attentional lapses are hard to accommodate within the commonly held view of the DMN as supporting off-task states^76–78^. Instead our study adds to a growing body of evidence that this system can make important contributions to external task processing^68,79–83^.

In conclusion, we found that dissociable patterns of activation and intrinsic connectivity in an MTG region within DMN predicted text-based memory and off-task thought. Better performance on questions about the text was associated with greater coupling of MTG with another DMN region in anterior cingulate gyrus. In contrast, greater disconnection between MTG and primary visual cortex was associated with frequent off-task thought. We conclude that DMN regions in lateral temporal cortex not only help us to process information in the external environment, but also form thoughts that can be independent from what is happening around us – however, both of these aspects of cognition are supported by a broader network of brain regions.
